# Supplementary material for: Asymmetric Synthesis of Quaternary Hydantoins via a Palladium-Catalyzed Aza-Heck Cyclization
Source: J Am Chem Soc. 2025 Nov 14;147(49):44692–8. doi: 10.1021/jacs.5c16022 (PMC12703750; doi:10.1021/jacs.5c16022)
Supplement: Supplementary file 2 [file ja5c16022_si_002.zip › All NMR FID Files/S15/S15_AllNMR/fluorine/pdata/1/email_TDI01073_7_1.pdf]

F19_32 CDCl3 /opt/nmrdata tdidowu 17

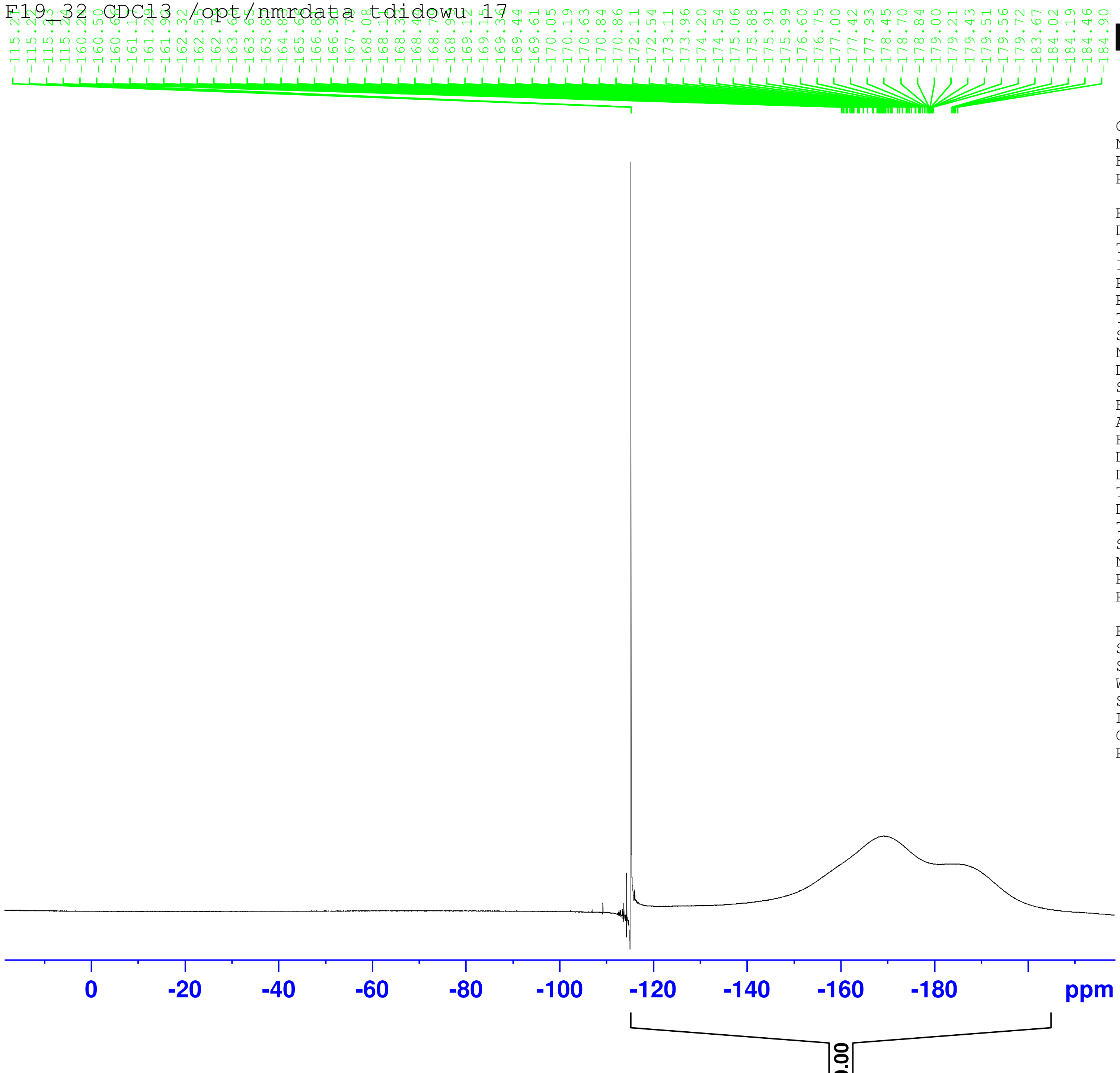

BRUKER

Current Data Parameters	
NAME	TDI01073
EXPNO	7
PROCNO	1
F2 - Acquisition Parameters	
Date_	20240208
Time	17.08 h
INSTRUM	spect
PROBHD	Z168773_0053 (
PULPROG	zgflqn
TD	131072
SOLVENT	CDCl3
NS	32
DS	4
SWH	133928.578 Hz
FIDRES	2.043588 Hz
AQ	0.4893355 sec
RG	10
DW	3.733 usec
DE	18.00 usec
TE	298.2 K
D1	1.00000000 sec
TD0	1
SFO1	564.8086803 MHz
NUC1	19F
P1	15.00 usec
PLW1	24.73399925 W
F2 - Processing parameters	
SI	65536
SF	564.8651668 MHz
WDW	EM
SSB	0
LB	0.30 Hz
GB	0
PC	1.00
